# Association of Body Mass Index With 21-Gene Recurrence Score Among Women With Estrogen Receptor–Positive, *ERBB2*–Negative Breast Cancer

**DOI:** 10.1001/jamanetworkopen.2022.43935

**Published:** 2022-11-28

**Authors:** Janghee Lee, Hakyoung Kim, Soong June Bae, Jung Hwan Ji, Jong Won Lee, Byung Ho Son, Sei Hyun Ahn, Joon Jeong, Sae Byul Lee, Sung Gwe Ahn

**Affiliations:** 1 Department of Surgery, Sacred Heart Hospital, Hallym University, Dongtan, Republic of Korea; 2 Department of Surgery, Asan Medical Center, University of Ulsan College of Medicine, Seoul, Republic of Korea; 3 Department of Surgery, Gangnam Severance Hospital, Yonsei University College of Medicine, Seoul, Republic of Korea; 4 Institute for Breast Cancer Precision Medicine, Yonsei University College of Medicine, Seoul, Republic of Korea

## Abstract

**Importance:**

Body mass index (BMI) may affect the 21-gene recurrence score (RS) in patients with ER-positive, *ERBB2*-negative breast cancer. If high BMI increases genomic risk in ER-positive, *ERBB2*-negative breast cancer, weight control will become more important.

**Objective:**

To assess the association between RS and BMI according to age groups and address BMI as a factor associated with high RS.

**Design, Setting, and Participants:**

This cohort study included 2295 patients with ER-positive, *ERBB2*-negative breast cancer who had undergone a multigene assay between March 29, 2010, and December 31, 2020, in 2 hospitals. All of the study patients were Korean women, and the median follow-up period was 45 months (range, 1-40 months). The correlations between continuous RS and BMI were investigated. A high BMI was defined as a body mass index greater than or equal to 25. In the younger age group (age ≤45 years), a high RS was defined as an RS of greater than 20.

**Exposures:**

Body mass index.

**Main Outcomes and Measures:**

The Pearson correlation coefficient was used to estimate the association between RS and BMI. A multivariable binary logistic model was used to identify high RS.

**Results:**

Among the 2295 women included (mean [SD] age, 49.8 [4.00] years; range, 22-81 years), 776 were aged 45 years or younger; RS and BMI were weakly correlated (correlation coefficient, 0.119; *P* < .001) in this younger group. Among them, the proportion of patients with an RS greater than 20 was significantly higher in the high BMI group than in the normal BMI group (45.5% [46 of 101] vs 27.3% [184 of 675]; *P* < .001). In the multivariable analysis, high BMI was an associated factor for high RS (odds ratio, 2.06; 95% CI, 1.28-3.32; *P* = .003). The 21-gene multigene assay-guided chemotherapy rate was significantly higher in patients with high BMI (30.7% [31 of 101] vs 20.2% [136 of 674]; *P* = .02).

**Conclusions and Relevance:**

In this cohort study of women aged 45 years or younger, high BMI was associated with higher RS in those with ER-positive, *ERBB2*-negative breast cancer; further studies are necessary to examine the underlying mechanisms.

## Key Points

**Question:**

Is high body mass index (BMI) a factor associated with a high 21-gene recurrence score in estrogen receptor (ER)–positive, *ERBB2*-negative (formerly *HER2* or *HER2/neu*) breast cancer, especially in younger patients (≤45 years) with breast cancer?

**Findings:**

In this cohort study, 776 women aged 45 years or younger with breast cancer had a weak positive correlation between 21-gene recurrence score and BMI. In these patients, high BMI was associated with a high recurrence score (>20).

**Meaning:**

The findings of this study suggest that increasing BMI might be associated with a higher genomic risk in younger patients with *ER+ERBB2−* breast cancer.

## Teaser

This cohort study assesses the correlation between body mass index (BMI) and the recurrence score in women with estrogen receptor (ER)–positive, *ERBB2*-negative breast cancer.

## Introduction

Body mass index (BMI; calculated as weight in kilograms divided by height in meters squared) is one of the most widely used indicators to measure obesity and is easily calculated based on body weight and height.^1^ A high BMI is associated with a worse prognosis in patients with estrogen receptor ER-positive, *ERBB2*-negative (formerly *HER2* or *HER2/neu*) breast cancer.^2,3,4,5^ In particular, an association between continuously increasing BMI and inferior survival outcomes was observed in 4770 patients with ER-positive, *ERBB2*-negative disease in the E1199 trial.^2^ Furthermore, obesity is a well-known risk factor for *ER+* breast cancer in women who are postmenopausal.^6,7,8^

This interaction between high BMI and *ER*+ breast cancer could be partly explained by an increase in estrogen biosynthesis from adipose tissues^9,10^ and contributes to the tumorigenesis and progression of breast cancer by creating a prooncogenic environment through the release of various cytokines,^11^ hypoxia-induced molecular change,^12^ and activation of inflammation.^13^

By contrast, in *ER+* breast cancer, the Oncotype Dx (Exact Sciences Corp) multigene assay is widely used to estimate the prognosis and guide the adjuvant treatment decisions.^14^ Intriguingly, because 5 genes of the ER module have lower recurrence scores (RSs) according to the algorithm,^15^ the RS from the assay becomes lower in endocrine-sensitive tumors, which might be affected by the estradiol levels. Otherwise, the proliferation module of RS could be upregulated by the obesity-related inflammation; it remains unclear whether the RS in women with a high BMI is lower or higher than in those with a normal BMI. A previous study showed that a higher BMI was associated with a lower RS in women who were postmenopausal with invasive lobular cancer,^16^ which is a principally hormone-driven cancer.^17,18^ A study by Pomponio et al^19^ suggested that the tumors of women who are postmenopausal with a higher BMI generally have a lower RS, whereas another study reported that the association of BMI with RS interacts with menopausal status, even though patients who are obese are more likely to have an RS less than 11 compared with that of women who are not overweight.^20^

We aimed to assess the association between continuous RS and BMI in 2295 patients who had undergone the Oncotype Dx multigene assay after primary surgery. Moreover, within the younger age group (≤45 years), we aimed to analyze whether BMI could be a factor associated with a high RS in these patients.

## Methods

### Study Population

This study followed the Strengthening the Reporting of Observational Studies in Epidemiology (STROBE) reporting guideline for observational studies. We included 2295 patients with primary invasive breast cancer who had undergone curative surgery at the Gangnam Severance Hospital and Asan Medical Center between March 29, 2010, and December 31, 2020. Our study was approved by the institutional review boards of Gangnam Severance Hospital and Asan Medical Center. The requirement of informed consent was waived by the institutional review boards due to the retrospective study design.

**Figure 1.**
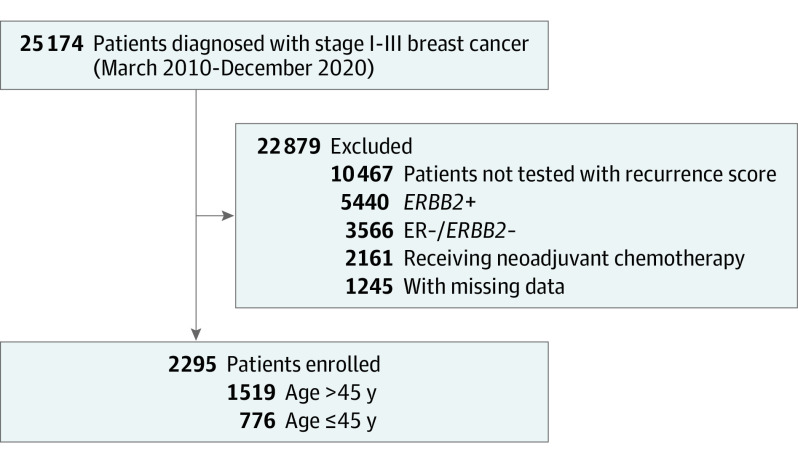
Diagram of Selecting Enrolled Patients ER indicates estrogen receptor; *ERBB2+*, estrogen-receptor–positive; *ERBB2*−, *ERBB2*-negative.

All patients were diagnosed with stage I to III ER-positive, *ERBB2*-negative breast cancer and had undergone a 21-gene RS (Oncotype DX) test using tumor tissue samples obtained from surgical specimens. Patients who did not undergo a 21-gene RS test or had *ER*-negative or *ERBB2*-positive breast cancer were excluded. Patients who received neoadjuvant chemotherapy or were diagnosed with de novo stage IV cancer were also excluded. In Figure 1, we summarize our study population. The clinicopathologic information of patients, including tumor-node metastasis stage, histologic grade, nuclear grade, progesterone receptor (PgR) status, and adjuvant treatments, were obtained retrospectively through a medical records review. Adjuvant endocrine or chemoendocrine treatments were determined based on the clinical and pathologic information and the RS. Patients treated for ovarian function suppression for at least 6 months were included in the ovarian function suppression group. The expression level of the Ki-67 labeling index (LI) was examined using immunohistochemistry (MIB-1; Dako) and presented as a percentage (range, 0%-100%) of positive tumor cells.

### Body Mass Index

The body weight and height were measured before initiation of the first treatment. All patients had undergone physical measurements at the first outpatient visit or upon admission for surgery. The period between the initiation of treatment and the measurement of body weight and height did not exceed 2 months. Body mass index was calculated as body weight in kilograms divided by height in meters squared, as defined by the World Health Organization (WHO).^1^ According to the WHO-Asia-Pacific classification, obesity in Asian individuals is defined as a BMI equal to or higher than 25.^21^ Because all of the study patients were Korean, they were classified into 2 groups: the high BMI group (≥25) and the normal BMI group (<25).

### RS by 21-Gene Multigene Assay and Clinical Risk

A 21-gene RS test was performed using tumor tissue samples obtained from the surgical specimens of all enrolled patients, as requested by the central laboratory of Genomic Health. The 21 RS genes consisted of 16 cancer-related genes (*MKI67*, *STK15*, *BIRC5*, *CCNB1*, *MYBL2*, *MMP11*, *CTSL2*, *GRB2*, *ERBB2*, *ER*, *PGF*, *BCL2*, *SCUBE2*, *GSTM1*, *CD68*, and *BAG1*) and 5 reference genes (*ACTB*, *GAPDH*, *RPLPO*, *GUS*, and *TFRC*). In patients with multiple tumors, the RS results from the largest tumor were used.

A previous study that conducted a subgroup analysis of the TAILORx trial reported that an RS of 20 considered as low clinical risk and an RS of 15 considered as high clinical risk could be the criteria for deciding to initiate chemotherapy in women who are premenopausal.^22^ Based on this result, all women with ER-positive, *ERBB2*-negative breast cancer who are premenopausal and have an RS greater than 20 are candidates for chemotherapy, regardless of the clinical risk. In the present study, the patients were divided into high (≥20) and low (<20) RS groups.

The exploratory subgroup analyses were performed; the association between BMI and RS was further explored according to the subgroups. In addition to the conventional risk factors, the clinical risk was addressed using the criteria applied in a previous study that validated the results of a multigene assay.^22,23^ The low clinical risk was defined as a tumor size of 3 cm or less and histologic grade I, tumor size of 2 cm or less and histologic grade II, and tumor size of 1 cm or less and histologic grade III in patients with lymph node (LN)-negative status and tumor size of 2 cm or less and histologic grade I in patients with LN-positive status. All patients who did not meet the classification for low clinical risk were considered to have high clinical risk.

### Statistical Analysis

The primary objective of this study was to determine the correlation between RS and BMI. Pearson correlation coefficient was used to estimate the correlation between the continuous RS and BMI. The correlation coefficients for each age group are displayed using a spline curve. The differences in the correlation coefficients of patients aged less than or equal to 45 years vs greater than 45 years were addressed using Fisher *Z* test. The 2-sided, paired interactions between RS, BMI, and age less than or equal to 45 years vs greater than 45 years were evaluated at a significance level of .05. Discrete variables were compared using the χ^2^ test. Continuous variables were compared using Mann-Whitney and Kruskal-Wallis tests. The *t* test or 1-way analysis of variance was used to compare the means. A binary logistic model was used for univariate and multivariate analysis to identify factors for high RS and subgroup analysis according to LN metastasis, clinical risk, PgR receptor, and Ki-67 LI. Variables with statistical significance in the univariate analysis were included in the multivariate model.

Recurrence-free survival (RFS) was defined as the period between breast cancer surgery and the recurrence of breast cancer. Recurrence events included locoregional recurrence in the ipsilateral breast or axillary LN and distant recurrence. Contralateral breast cancer was considered a secondary malignancy and excluded from the recurrence event. The Kaplan-Meier survival curve was used for RFS analysis. All statistical analyses were performed using R software, version 3.6.1 (R Foundation for Statistical Computing) and GraphPad Prism, version 8 (GraphPad Software). A *P* value <.05 was considered significant.

## Results

### Study Population

A total of 2295 patients from 2 hospitals were included (mean [SD] age, 49.8 [4.00] years; range, 22-81 years) (eTable in the Supplement). Of these, 1708 patients (74.4%) had a low BMI (<25), whereas 587 patients (25.6%) had a high BMI (≥25). The mean (SD) age of the low BMI group (48.4 [8.77] years) was younger than that of the high BMI group (54.0 [9.44] years). Similarly, the proportion of patients with premenopausal status was higher in the low vs high BMI group (1151 [67.4%] vs 258 [44.0%]). The 21-gene assay was performed successfully in all patients, and the RS was not different between the 2 groups stratified by BMI. With regard to other clinical and pathologic parameters, the high BMI group was likely to have larger tumors. In addition, significantly higher rates were noted in the high vs low BMI groups for PgR-negative (62 [10.6%] vs 129 [7.6%]; *P* = .02) and low Ki-67 LI (339 [57.8%] vs 1085 [63.5%]; *P* = .01).

### Correlation Between RS and BMI in the Younger Age Group (≤45 Years)

**Figure 2.**
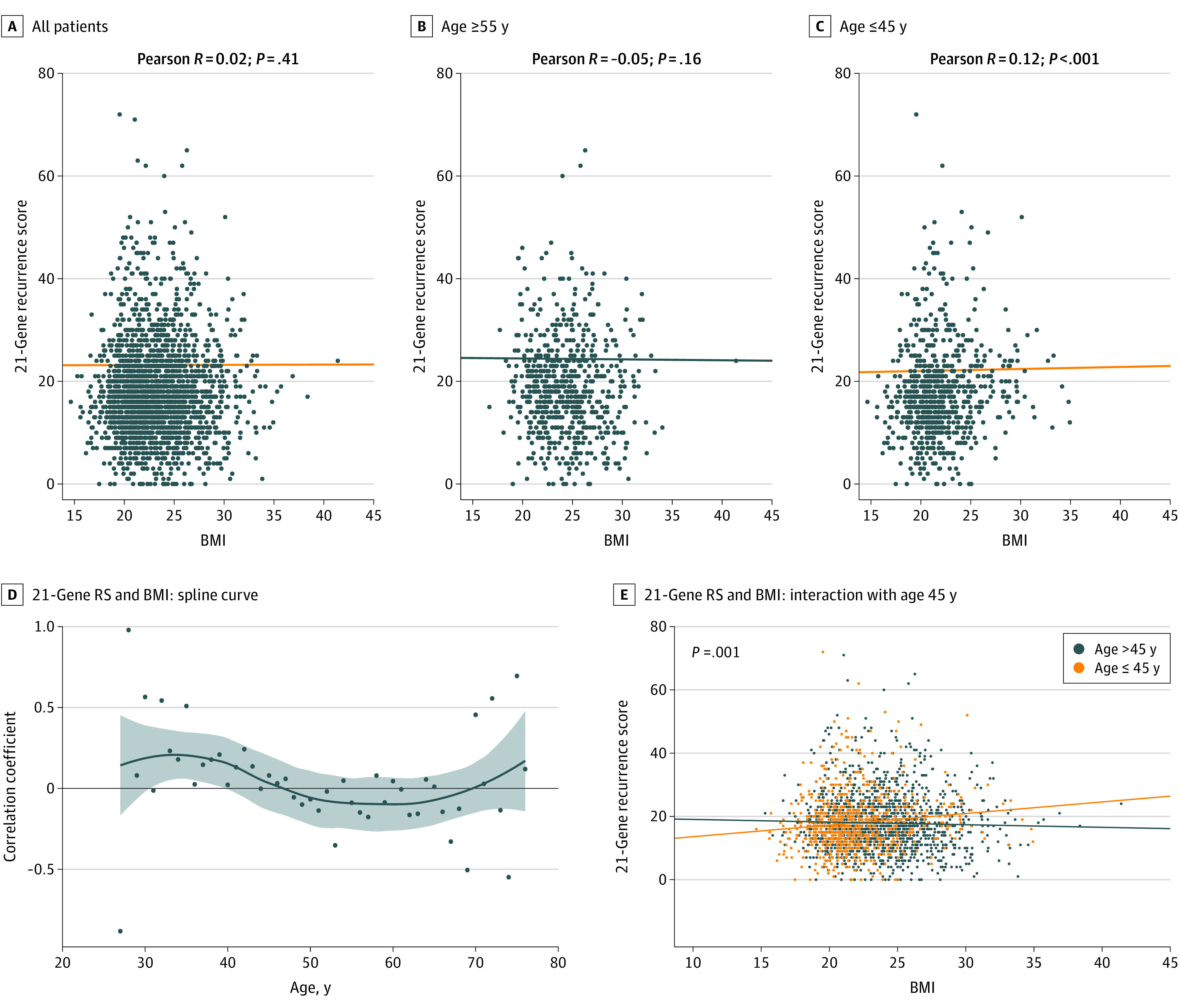
Correlation of Body Mass Index (BMI) and 21-Gene Recurrence Score With Correlation Coefficients Estimated Using Pearson Method A, All patients (N = 2295). B, Patients aged 55 years or older (n = 661). C, Patients aged 45 years or younger (n = 776). D, Spline curve of the correlation coefficient between BMI and 21-gene RS according to age. E, An interaction effect of age 45 years and the correlation between BMI and 21-gene RS. Body mass index is calculated as weight in kilograms divided by height in meters squared.

First, we investigated the correlation between continuous BMI and RS (Figure 2A). No correlation was observed between the 2 continuous parameters (correlation coefficient = 0.02; *P* = .41). However, a very weak inverse correlation was found between the 2 parameters in women who were postmenopausal (correlation coefficient = −0.08; *P* = .02), whereas these 2 parameters were very weakly correlated in women who were premenopausal (correlation coefficient = 0.05; *P* = .050). To minimize the influence of perimenopause, the patients were divided into the following groups: those aged 55 years or older (older age group) and those aged 45 years or younger (young age group). In the older age group (n = 661), the inverse correlation between RS and BMI was not significant (correlation coefficient = −0.05; *P* = .16) (Figure 2B). Among the younger aged group (n = 776), RS and BMI were weakly correlated with a statistical significance (correlation coefficient = 0.12; 95% CI, 0.05-0.19; *P* < .001) (Figure 2C).

When a spline curve was constructed to interpolate the correlation coefficients in each age group, the correlation coefficients switched from positive to negative at approximately age 45 years (Figure 2D). To evaluate the difference between the 2 correlation coefficients of RS and BMI according to age (45 years), Fisher *Z* test was applied, and a significant difference was observed (comparison *P* < .001). In addition, when a linear regression model was performed to investigate an interaction effect in the correlation between RS and BMI according to age, the correlation between RS and BMI was significant in relation to age 45 years (*P* = .001 for interaction) (Figure 2E). Hence, further analyses were performed on the younger age group.

### RS and BMI in the Younger Age Group

**Figure 3.**
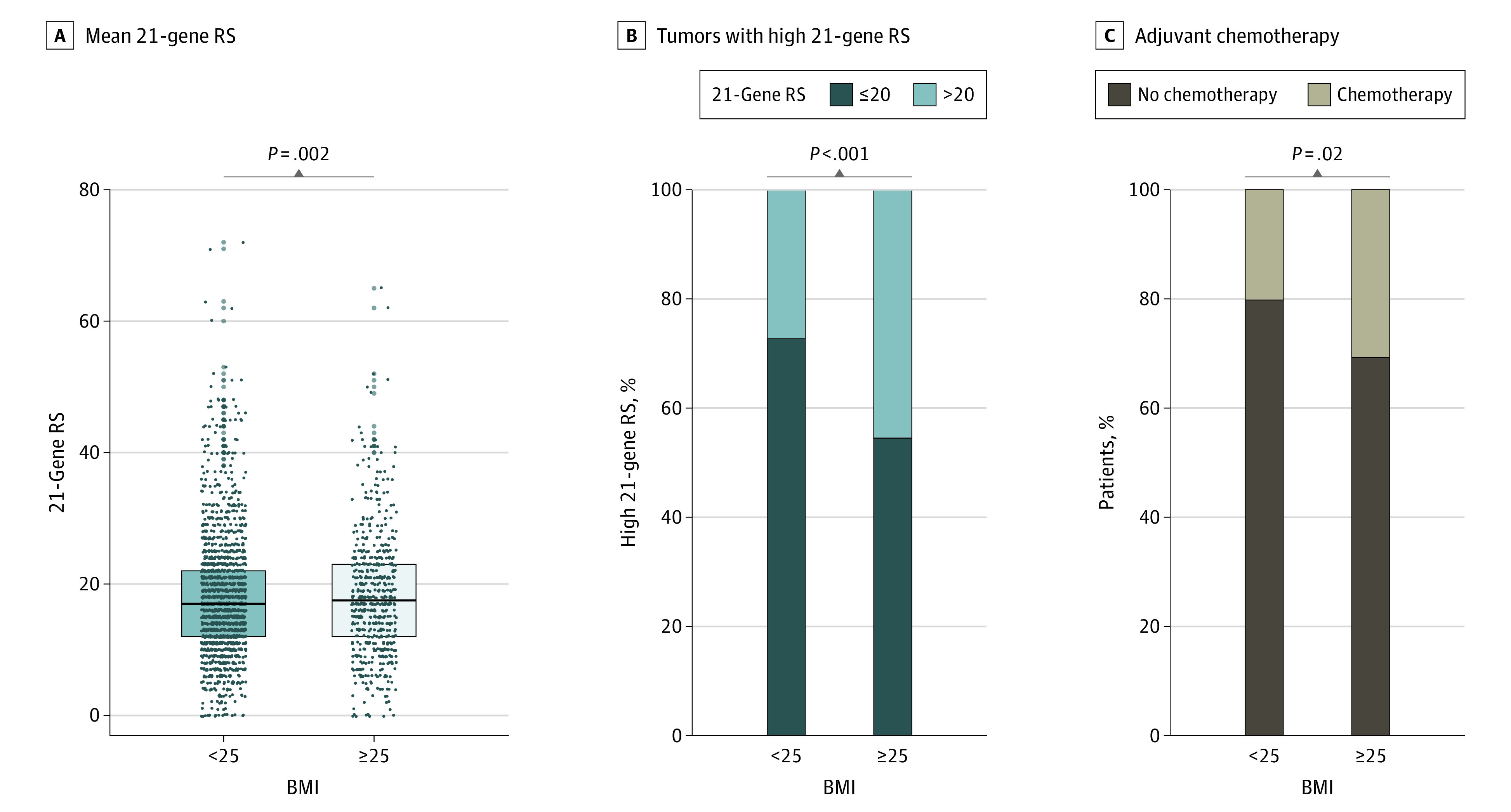
The 21-Gene Recurrence Score (RS) and Adjuvant Chemotherapy Rate According to Body Mass Index (BMI) in Patients Aged 45 Years or Younger A, Means of 21-gene RS were significantly higher in the high BMI group than in the low BMI group (mean [SE], 20.6 [0.86] vs 17.6 [0.35]; *P* = .002). Center lines indicate mean value of RS according to BMI, and dots indicate the RS of each patients. B, The rate of tumors with high 21-gene RS was significantly higher in the high BMI group than in the low BMI group (45.5% vs 27.3%; *P* < .001). C, The rate of adjuvant chemotherapy was significantly higher in the high BMI group than in the low BMI group (30.7% vs 20.2%; *P* = .02). The data on 1 patient in the low BMI group are missing. Body mass index is calculated as weight in kilograms divided by height in meters squared.

The clinical and pathologic characteristics were compared according to the BMI level in the younger age group (Table 1). Almost all patients were premenopausal regardless of the BMI group. The breast-conserving surgery incidence was higher in the high BMI group than in the low BMI group. When the mean RS was compared according to the BMI level, it was significantly higher in the high BMI group (20.6% vs 17.6%; *P* = .001) (Figure 3A). In addition, the proportion of patients with an RS of greater than 20 was significantly higher in the high BMI group than in the normal BMI group (45.5% [46 of 101] vs 27.3% [184 of 675]; *P* < .001) (Figure 3B).

**Table 1.** Comparison of Clinicopathologic Characteristics According to BMI Level in Patients Aged 45 Years or Younger

Variable	No. (%)		*P* value
	BMI <25 (n = 675)	BMI ≥25 (n = 101)	
21-gene RS, mean [SE] (range)	17.6 [0.35] (0-72)	20.6 [0.86] (0-52)	.002
Menopausal status			
Premenopausal	669 (99.1)	101 (100)	.64
Postmenopausal	5 (0.7)	0	
Unknown	1 (0.1)	0	
Breast surgery type			
BCS	462 (68.4)	79 (78.2)	.05
Mastectomy	213 (31.6)	22 (21.8)	
Tumor size, mm			
≤20	442 (65.5)	58 (57.4)	.12
>20	233 (34.5)	43 (42.6)	
LN metastasis			
Negative	544 (80.6)	76 (75.2)	.21
Positive	131 (19.4)	25 (24.8)	
PgR			
Negative	25 (3.7)	4 (4.0)	.90
Positive	650 (96.3)	97 (96.0)	
Histologic grade			
I	71 (10.5)	9 (8.9)	.02
II	533 (79.0)	71 (70.3)	
III	68 (10.1)	20 (19.8)	
Unknown	3 (0.4)	1 (1.0)	
Nuclear grade			
I	17 (2.5)	2 (2.0)	.07
II	571 (84.6)	77 (76.2)	
III	84 (12.4)	21 (20.8)	
Unknown	3 (0.4)	1 (1.0)	
Ki-67 LI (%)			
<20	424 (62.8)	54 (53.5)	.07
≥20	251 (37.2)	47 (46.5)	

Abbreviations: BCS, breast-conserving surgery; BMI, body mass index (calculated as weight in kilograms divided by height in meters squared); LI, labeling index; LN, lymph node; PgR, progesterone receptor; RS, recurrence score.

### Association of High BMI and High RS in the Younger Age Group

In the univariate analyses, high BMI was an associated factor for high RS in the younger age group (odds ratio [OR], 2.28; 95% CI, 1.40-3.73; *P* = .001). Age, PgR-negative status, high histologic grade, and high Ki-67 LI were also related factors for high RS. In the multivariable analysis adjusted for other factors (Table 2), BMI was an independent factor for RS (OR, 2.06; 95% CI, 1.28-3.32; *P* = .003).

**Table 2.** Univariate and Multivariate Analyses of Factors Affecting the 21-Gene RS in Patients Aged 45 Years or Younger

Variable	Univariate analysis		Multivariate analysis	
	OR (95% CI)	*P* value	OR (95% CI)	*P* value
Age^a^	0.93 (0.90-0.96)	<.001	0.95 (0.91-0.98)	.004
BMI				
<25.0	1 [Reference]	NA	1 [Reference]	NA
≥25.0	2.28 (1.40-3.73)	.001	2.06 (1.28-3.32)	.003
Breast surgery type			NA	
BCS	1 [Reference]	NA	NA	NA
Mastectomy	0.78 (0.53-1.15)	.21	NA	NA
Tumor size, mm				
≤20	[Reference]	NA	NA	NA
>20	1.35 (0.95-1.93)	.10	NA	NA
LN metastasis				
Negative	1 [Reference]	NA	NA	NA
Positive	1.03 (0.70-1.51)	.88	NA	NA
PgR				
Negative	1 [Reference]	NA	1 [Reference]	NA
Positive	0.09 (0.04-0.26)	<.001	0.09 (0.04-0.23)	<.001
Histologic grade				
I	1 [Reference]	NA	1 [Reference]	NA
II	2.21 (1.02-4.78)	.05	2.07 (0.99-4.31)	.05
III	12.00 (4.93-29.20)	<.001	8.61 (3.364-20.40)	<.001
Nuclear grade				
I	1 [Reference]	NA	NA	NA
II	NE	NA	NA	NA
III	NE	NA	NA	NA
Ki-67 LI (%)				
<20	1 [Reference]	NA	1 [Reference]	NA
≥20	3.25 (2.36-4.48)	.001	2.35 (1.65-3.36)	<.001

Abbreviations: BCS, breast-conserving surgery; BMI, body mass index (calculated as weight in kilograms divided by height in meters squared); LI, labeling index; LN, lymph node; NA, not applicable; NE, not estimated; OR, odds ratio; PgR, progesterone receptor; RS, recurrence score.

^a^ Continuous variable.

In addition, the adjuvant treatments were compared according to the BMI level. The adjuvant radiotherapy rate in the high BMI group was higher as the breast-conserving surgery rate was higher in these groups, although the difference was not significant (70.4% [475 of 675] vs 79.2% [80 of 101]; *P* = .07). In addition, patients with a high BMI were more likely to receive ovarian function suppression–based endocrine therapy than those with a low BMI (43.7% [295 of 675] vs 53.5% [54 of 101]; *P* = .07). In particular, the incidence of 21-gene multigene assay-guided chemotherapy was also significantly higher in patients with a high BMI (30.7% [31 of 101] vs 20.2% [136 of 674]; *P* = .02) (Figure 3C).

At a median follow-up of 45 months (range, 1-140), 33 patients experienced cancer recurrence, and the 5-year RFS was 96.8%. In the survival analyses using the Kaplan-Meier survival estimate, RFS did not differ according to BMI, but it differed significantly according to RS (3.1% [17 of 546] vs 7.0% [16 of 230]; hazard ratio, 2.31; 95% CI, 1.17-4.58; *P* = .01) (eFigure A and B in the Supplement).

### Subgroup Analysis of BMI and High 21-Gene RS

We further performed exploratory subgroup analyses. The association between BMI and RS was investigated according to subgroup. In the subgroup analyses, high BMI was a significant associated factor in the subgroups with low clinical risk (odds ratio [OR], 2.36; 95% CI, 1.20-4.65) or high clinical risk (OR, 2.00; 95% CI, 1.14-3.51), PgR+ (OR, 2.59; 95% CI, 1.67-4.00), and high Ki-67 LI (OR, 2.49; 95% CI, 1.31-4.76) (eFigure C in the Supplement). In the PgR-negative group (n = 29), low BMI was associated with high RS. In addition, high BMI was a factor associated with high RS in the LN-negative subgroup but not in the node-positive subgroup; however, the ORs were similar between the 2 subgroups (eFigure C in the Supplement).

## Discussion

Our observational study noted a weakly positive correlation between BMI and RS in patients with ER-positive, *ERBB2*-negative breast cancer aged 45 years or younger. Among these patients, high BMI was an independent factor for high RS. Therefore, luminal cancer that develops in young women with a high BMI is likely to have a high genomic profile. As a result, this association between BMI and RS led to an increased proportion of patients with a high BMI receiving chemotherapy than those with a low BMI, although RFS did not differ substantially according to BMI level. To our knowledge, this study is the first to report the association between BMI and RS among younger patients with ER-positive, *ERBB2*-negative breast cancer.

Previous studies have suggested that high BMI is associated with low RS in women who are postmenopausal.^16,19^ In addition, researchers interpreted this correlation based on the theory that enriched adipocytes in women who are obese increase the plasma levels of estradiol and progesterone,^24,25^ which may curtail the RS by promoting ER and PgR expression in tumors. By contrast, our findings showed an association between high BMI and RS in younger women who were premenopausal. To explain the association between BMI and RS in younger women in our study, it is unreasonable to focus only on the effects of sex hormones, which are already abundant in younger women who are premenopausal. Since RS is altered not only by the genes in ER module but also by those in the proliferation module in women who are premenopausal, the RS might be largely influenced by obesity-associated proinflammatory cytokines than by obesity-associated elevated serum sex hormonal levels.

A study by Tong et al^20^ investigating the associations between each single gene of RS and BMI reported that a high BMI could increase the expression of proliferation-related genes, such as *Ki-67* and *CCNB1*, as well as ER-related genes such as *ER*, *PR*, and *CEGP1*. This finding supports the results of our study because it provides external evidence noting that high BMI may increase the RNA expression of proliferation-related genes in RS. In our study, the high Ki-67 LI rate in patients with high BMI (Table 1), although not significant, also supports this hypothesis.

Obesity promotes the secretion of proinflammatory cytokines, resulting in inflammation.^26^ An experimental study reported that interleukin (IL)-6 derived from adipose stromal cells stimulates migration and invasion of malignant breast cells.^27^ In addition, this study showed that adipocytes promote the invasive capacity of cancer cells in *ER−* and *ER+* breast cancer, indicating that this mechanism is independent of the hormonal function of adipose tissue. Other studies have reported that high serum levels of circulating IL-6 negatively impact the RFS and are associated with poor prognosis in patients with breast cancer.^28,29^ Taken together, further studies are warranted to understand the complexity of the mechanism by which enriched adipose tissue affects the genomic profiles of young patients with *ER+* breast cancer with obesity.

In the exploratory subgroup analysis, BMI was a factor related to high RS in the groups with aggressive features, such as high clinical risk or high Ki-67 LI (eFigure C in the Supplement). Since the secondary analysis of the TAILORx trial showed estimates of the absolute chemotherapy benefit (mean [SD] 8.7 [6.2] percentage points) in the high-risk group with an RS of 21 to 25,^22^ chemotherapy is strongly recommended. In the younger age group with high clinical risk, the RS rate was significantly higher (50.8% in the high BMI group and 34.1% in the low BMI group; *P* = .01), and the chemotherapy rate tended to be higher in the high BMI group (37.3%) than in the low BMI group (27.9%) (*P* = .15). Thus, BMI is clinically important as a factor associated with high RS in the group with high clinical risk.

### Limitations

This study has limitations; the major limitation is its retrospective nature. In particular, our study population received RS-guided adjuvant treatments; hence, it is difficult to discover the survival difference according to BMI level. Another reason for the lack of survival difference based on BMI in younger women is that the RS of women with high BMI ranges from 21 to 25, which is not higher than 25 (RS 21-25: 82.2%; RS >25: 17.8%). Although, to our knowledge, this was the largest study that focused on younger patients and performed multigene assays, our cohort only consisted of Korean women. The proportion of patients with obesity varies depending on race and ethnicity. Moreover, because the proportion of risk classification by RS and its prognostic influence may differ by race and ethnicity,^30^ our findings with Korean women cannot be generalized to populations of other geographic regions.^31^ Therefore, it is worthwhile to explore whether the association of BMI with RS varies by race and ethnicity in future research.

## Conclusions

In this observational study, high BMI was an independent factor associated with high RS in younger patients (≤45 years). Our findings suggest that increasing BMI might be associated with a higher genomic risk in younger patients with ER-positive, *ERBB2*-negative breast cancer. Further studies are necessary to dissect the complexity of effects of obesity on genomic profiles of patients with *ER+* breast cancer.

## Notes

Supplement. **eFigure.** Kaplan-Meier Survival Curve of RFS and Subgroup Analysis in Patients Aged ≤45 Years **eTable.** Baseline Characteristics According to BMI Level in All Patients
