# Supplementary material for: Association of Body Mass Index With 21-Gene Recurrence Score Among Women With Estrogen Receptor–Positive, ERBB2–Negative Breast Cancer
Source: JAMA Netw Open. 2022 Nov 28;5(11):e2243935. doi: 10.1001/jamanetworkopen.2022.43935 (PMC9706366; doi:10.1001/jamanetworkopen.2022.43935)
Supplement: Supplement. — eFigure. Kaplan-Meier Survival Curve of RFS and Subgroup Analysis in Patients Aged ≤45 Years eTable. Baseline Characteristics According to BMI Level in All Patients [file jamanetwopen-e2243935-s001.pdf]

Supplementary Online Content

Lee J, Kim H, Bae SJ, et al. Association of body mass index with 21-gene recurrence score among women with estrogen receptor-positive, *ERBB2*-negative breast cancer. *JAMA Netw Open*. 2022;5(11):e2243935. doi:10.1001/jamanetworkopen.2022.43935

eFigure. Kaplan-Meier Survival Curve of RFS and Subgroup Analysis in Patients Aged ≤45 Years

eTable. Baseline Characteristics According to BMI Level in All Patients

This supplementary material has been provided by the authors to give readers additional information about their work.

© 2022 Lee J et al. *JAMA Network Open.*

eFigure. Kaplan-Meier survival curve of RFS and subgroup analysis in patients aged ≤45 years.

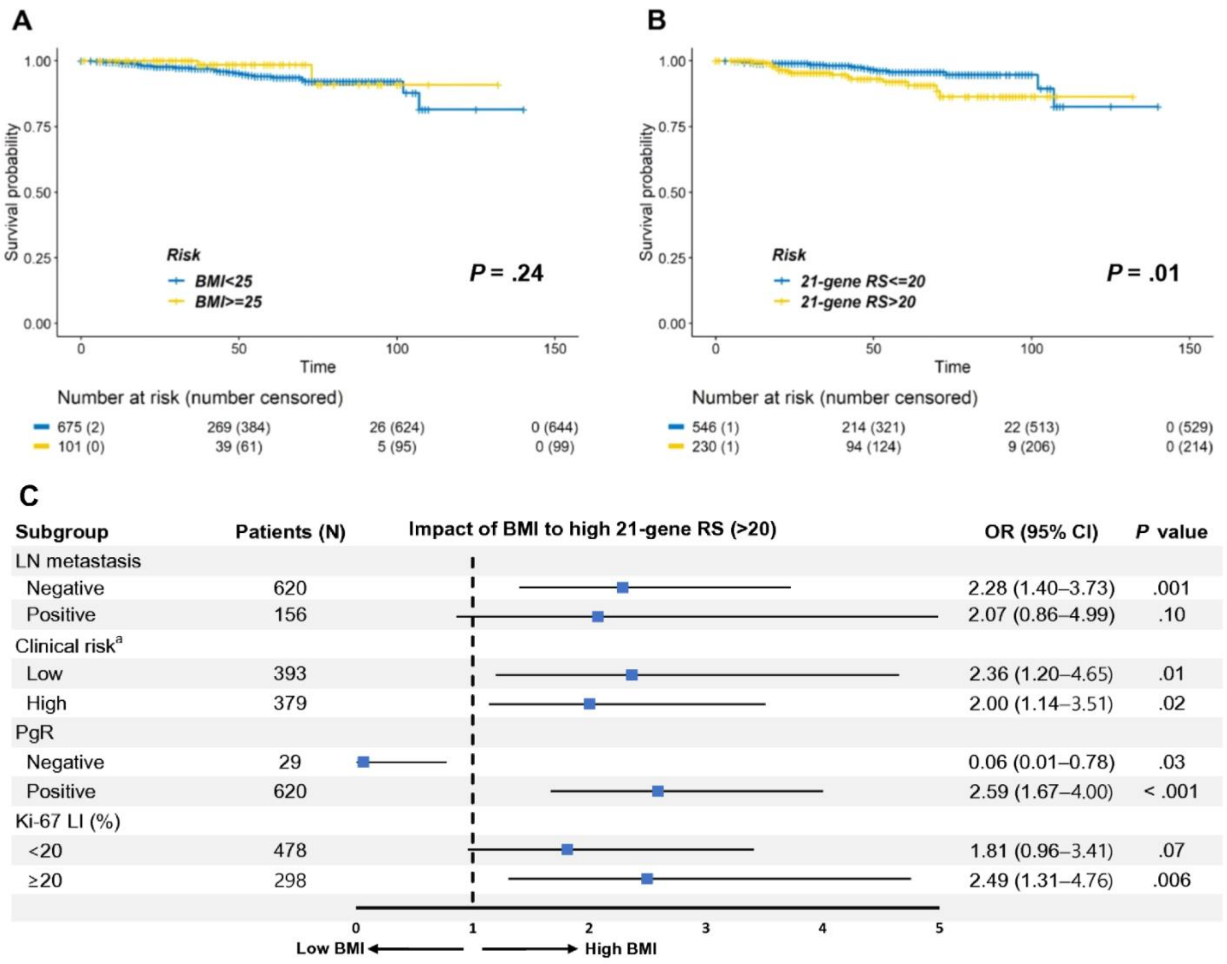

(A) Comparison according to BMI level (*P* = .24). (B) Comparison according to 21-gene RS (*P* = .01). (C) Forest plot of the association between BMI and high 21-gene RS

[a]Low clinical risk: tumor size of ≤3 cm and HG I, tumor size of ≤2 cm and HG II, and tumor size of ≤1 cm and HG III in patients who were LN-negative; tumor size of ≤2 cm and HG I in patients who were LN-positive

RFS, recurrence-free survival; BMI, body mass index; RS, recurrence score; OR, odds ratio; LN, lymph node; PgR, progesterone receptor; LI, labeling index; HG, histologic grade

© 2022 Lee J et al. *JAMA Network Open.*

eTable. Baseline characteristics according to BMI level in all patients

	All patients (%) (N=2,295)	BMI<25kg/m^2 (%) (N=1,708)	BMI≥25kg/m^2 (%) (N=587)	*P* value
Age, mean (range)	49.8 (22–81)	48.4 (22–81)	54.0 (28–78)	< .001
Menopausal status				< .001
Premenopausal	1,409 (61.4)	1,151 (67.4)	258 (44.0)	
Postmenopausal	809 (35.3)	502 (29.4)	307 (52.3)	
Unknown	77 (2.3)	55 (3.2)	22 (3.7)	
21–gene RS, mean (range)	17.9 (0–72)	17.9 (0–72)	18.1 (0–65)	.64
Breast surgery type				.09
BCS	1,726 (75.2)	1,269 (74.3)	457 (77.9)	
Mastectomy	569 (24.8)	439 (25.7)	130 (22.1)	
Tumor size (mm)				< .001
≤20	1,481 (64.5)	1,144 (67.0)	337 (57.4)	
>20	814 (35.5)	564 (33.0)	250 (42.6)	
LN metastasis				.45
Negative	1,862 (81.1)	1,392 (81.5)	470 (80.1)	
Positive	433 (18.9)	316 (18.5)	117 (19.9)	
PgR				.02
Negative	191 (8.3)	129 (7.6)	62 (10.6)	
Positive	2,104 (91.7)	1,579 (92.4)	525 (89.4)	
Histologic grade				.18
I	210 (9.2)	158 (9.3)	52 (8.9)	
II	1,854 (80.8)	1,392 (81.5)	462 (78.7)	
III	225 (9.8)	154 (9.0)	71 (12.1)	
Unknown	6 (0.3)	4 (0.2)	2 (0.3)	
Nuclear grade				.22
I	55 (2.4)	41 (2.4)	14 (2.4)	
II	1,956 (85.2)	1,469 (86.0)	487 (83.0)	
III	278 (12.1)	193 (11.3)	85 (14.5)	
Unknown	6 (0.3)	5 (0.3)	1 (0.2)	
Ki-67 LI (%)				.01
<20	1,424 (62.0)	1,085 (63.5)	339 (57.8)	
≥20	871 (38.0)	623 (36.5)	248 (42.2)	

BMI, body mass index; RS, recurrence score; BCS, breast-conserving surgery; LN, lymph node; PgR, progesterone receptor; HER2, human epidermal growth factor receptor 2; LI, labeling index

© 2022 Lee J et al. *JAMA Network Open.*
